# Convective Heat and Mass Transport in Casson Fluid Flow in Curved Corrugated Cavity with Inclined Magnetic Field

**DOI:** 10.3390/mi13101624

**Published:** 2022-09-28

**Authors:** Imtiaz Ali Shah, Sardar Bilal, Muhammad Imran Asjad, ElSayed M. Tag-ElDin

**Affiliations:** 1 Department of Mathematics, AIR University, Sector E-9, Islamabad 44000, Pakistan; 2 Department of Mathematics, University of Management and Technology, Lahore 54000, Pakistan; 3 Faculty of Engineering and Technology, Future University in Egypt, New Cairo 11835, Egypt

**Keywords:** convective thermosolutal transport, Casson fluid, curved corrugated enclosure, MHD, FEM

## Abstract

Convection in fluids produced by temperature and solute concentration differences is known as thermosolutal convection. It has valuable utilization in wide industrial and technological procedures such as electronic cooling, cleaning, and dying processes, oxidation of surface materials, storage components, heat exchangers, and thermal storage systems. In view of such prominent physical significance, focus is made to explicate double (thermal and solutal)-diffusive transport in viscoelastic fluid characterized by the Casson model enclosed in a curved enclosure with corrugations. An incliningly directed magnetic field is employed to the flow domain. A uniformly thermalized and concentrated circular cylinder is installed at the center of the enclosure to measure transport changes. Dimensionally balanced governing equations are formulated in 2D, representing governed phenomenon. Finite element-based open-sourced software known as COMSOL is utilized. The domain of the problem is distributed in the form of triangular and quadrilateral elements. Transport distributions are interpolated by linear and quadratic polynomials. The attained non-linear system is solved by a less time and computation cost consuming package known as PARDISO. Convergence tests for grid generation and validation of results are executed to assure credibility of work. The influence of involved physical parameters on concerned fields are revealed in graphical and tabular manner. Additionally, heat and mass fluxes, along with, kinetic energy variation are also evaluated.

## 1. Introduction

The bulk movement of liquids due to diversified distribution of densities is known as convection, and the joint effect of such variations on thermal and solutal behavior of involved material is termed thermosolutal convection. The pervasive utility of dual (thermal and solutal) diffusion is evidenced in various industrial and environmental processes, e.g., nuclear waste storage, oceanography, solidification and petrochemical processes, removal of contaminants from water, purification of air from pollutants, and so forth. The capitalization of double-diffusive transport in different procedures is also evidenced in refs. [1,2,3,4,5,6,7,8,9,10,11]. Recently, a recommendable survey has been commenced to evaluate diffusive transport in multiple flow problems, for instance convective current generated in air by opposing and assisting buoyancy driven forces was elucidated by Beghein et al. [12]. The effectiveness of buoyancy forces on solutal and energy transmission was comprehendingly disclosed by Gobin and Benacaer [13]. The influence of inertial forces on thermal and solutal diffusions by providing mixed convection to water in closed configuration was manifested by Amiri et al. [14]. Nithyadevi and Yang [15] examined the generation of flux in heat and mass distributions in an enclosure with provision of isothermal and concentration distributions at extremities. Numerical simulations were executed to determine change in heat and mass transfer by providing thermally buoyant flow in a vertical annulus by Chen et al. [16]. A finite difference scheme was utilized by Qin et al. [17] to adumbrate convective transport with diffusion aspects due to inertial and potential temperature differences. Some outstanding works quantifying diffusive thermosolutal transport are enclosed in refs. [18,19,20,21,22,23]. To highlight the behavior of critical shear-dependent materials, fluid models have been proposed. In this regard, the Casson fluid was proposed by Casson in [24], which is assumed to be the fittest one because it illuminates viscosity behavior at infinite shear stress. Viscoelastic liquids are given more physical significance because they depict the properties of blood. So, characterizing the flow of blood in arteries utilizing the Casson model is preferred. For the last 20 years, the Casson model has been taken into account in different computational domains and in various physical circumstances. Likewise, Rao et al. [25] determined physical and mathematical aspects to restrict Casson fluid to Newtonian by providing surface drag then yield stress. Kashif et al. [26] executed the unsteady flow of Casson fluid characterized by stretchable surface with mass and heat transmission. Ali et al. [27] revealed the magnetized flow of Casson liquid with the insertion of nanoparticles in a horizontally placed cylinder. The electroosmotic flow of Casson liquid in a channel with sinusoidal boundaries was scrutinized by Saleem et al. [28]. For the sake of readers’ interest, some topical work has been cited in refs. [29,30,31,32,33,34,35].

For restricting flow in a laminar regime, the best technique is the application of a magnetic field in transversal direction. The interaction of flow distributions with a magnetic field engenders a new class of liquids called electrically conducting fluids. These magnetized fluids play vital roles in numerous engineering disciplines. Even the joint execution of magnetization with heat transfer will make it more effective in producing stratified environments for producing or reducing the temperature of systems. Some remarkable utilizations of magnetic fluid are seen in fusion/fission reactors, solar technology, crystal growth, therapies, targeted drug delivery, and so many others. The magnetically influenced flow of viscous fluid in confined geometry along with heat transfer was conducted by Garandet et al. [36]. Rudraiah et al. [37] estimated the declining aptitude in flow distributions and opposing aspects of magnetic force in flow and uplift in heat flux rate. The dimensionless Hartmann number for flow in confined configurations by employing scaling of parameters was manifested by Hadid et al. [38]. Piazza and Ciofalo [39] considered a wide variation in Hartmann numbers from 100 to 10,000 and measured depreciation in velocity and uplift in heat and mass distributions. It is also observed that skin friction at the surface also enhances versus the Hartmann number. The unsteady MHD flow of 2D viscous fluid among two orthogonally permeable plates was commenced by Kashif et al. [40]. Bayones et al. [41] delineated the magnetic flow of stagnant fluid over a stretched surface by performing analytic and numerical solutions. Kashif et al. [42] examined the Soret and Dufour effects, first-order chemically, reacting Maxwell fluid past a stretched sheet concentrated, in a porous medium, and the series solution of magnetohydrodynamics. The performance of nanoparticles’ addition in base liquid enclosed in a wavy cavity in the presence of a magnetic field was divulged by Sheremet [43]. Khan [44] has made an effort to enlighten the heat and mass transfer using a first-order chemical reaction incorporating the magnetic effect in a boundary-layer MHD stagnation point flow on an elastic sheet through a porous medium. Hussain et al. [45] probed influence of magnetic field on thermal and solutal convective flow of Casson fluid in an enclosure by performing finite element computations. 

The effectiveness of thermosolutal convection in the above-mentioned scientific review acknowledges its importance. Although, for the prominence of this effort, it has been utilized in indoor temperature management, building construction designs, efficient automobile engines working, maintenance of shafts, and food processing. Additionally, the considered versatile fluid (Casson) in the current effort also raises the importance of study due to diversified and unique applications (paint industry, blood circulation, and emulsion formations). So, as far as the novelty of this article is concerned, this work represents its originality in the following ways:

1. The consideration of thermosolutal convection combinedly.
2. Considering curved corrugate domain to raise thermal performance of cavity.
3. Incorporation of Casson fluid in the study to perform the study in a more realistic approach.
4. The inclusion of inclined magnetic field effects instead of transversal ones.

So, in the authors’ opinion, this work is enriched with novelty and will definitely provide direction to researchers to work in this direction by considering different fluids and changing the design and structure of enclosures. It is worthwhile to mention that previously research has been performed with Newtonian fluids and enclosures without corrugations, so this will immensely assist researchers in exploring thermosolutal convection in complexed domains.

## 2. Mathematical, Modeling

### 2.1. Problem Formulation

**Figure 1:**
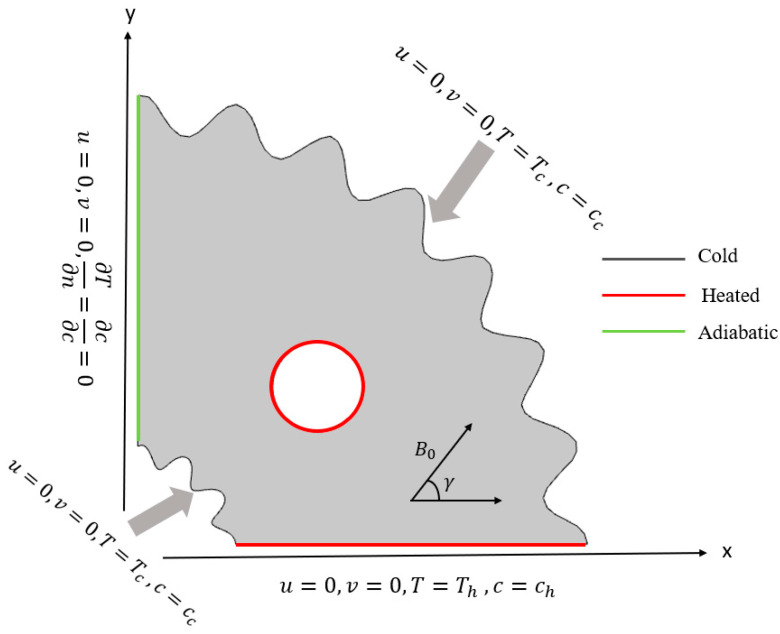
Graphical visualization of the domain.

We took into consideration the two-dimensional, steady, incompressible, and laminar flow of Casson liquid inside a curved corrugated cavity with a middle circular cylinder. The Boussinesq technique was used to formulate fluid density, taking into account shear-rate-dependent viscosity and the assumption that the fluid density is impersistent. At the bottom extremity and surface of the circular cylinder, uniform temperature $\left(T_{h}\right)$ and concentration $\left(C_{h}\right)$ are provided, whereas the curved surfaces are provided by cooled temperature $(T_{c}$) and constant concentration $\left(C_{c}\right)$. The vertical wall of the cavity is kept insulated. The inclined, magnetic, field of strength $B_{0}$ is employed, to the domain-making, angle of γ. Figure 1 presents a schematic illustration of a domain.

### 2.2. Governing, Equationss

The governing equations in dimensional form based on the aforementioned assumptions are as follows [45]:

(1) $$\;\;\;\frac{\partial u}{\partial x}+\frac{\partial v}{\partial y}=0,$$

(2) $$\rho \left(u\frac{\partial u}{\partial x}+v\frac{\partial u}{\partial y}\right)=-\frac{\partial p}{\partial x}+\mu \left(1+\frac{1}{\beta }\right)\left(\frac{\partial^{2}u}{\partial x^{2}}+\frac{\partial^{2}u}{\partial y^{2}}\right)+\Lambda_{x},$$

(3) $$\rho \left(u\frac{\partial v}{\partial x}+v\frac{\partial v}{\partial y}\right)=-\frac{\partial p}{\partial x}+\mu \left(1+\frac{1}{\beta }\right)\left(\frac{\partial^{2}v}{\partial x^{2}}+\frac{\partial^{2}v}{\partial y^{2}}\right)+\Lambda_{y},$$

(4) $$u\frac{\partial T}{\partial x}+v\frac{\partial T}{\partial y}=\alpha_{e}\left(\frac{\partial^{2}T}{\partial x^{2}}+\frac{\partial^{2}T}{\partial y^{2}}\right),\;$$

(5) $$u\frac{\partial T}{\partial x}+v\frac{\partial T}{\partial y}=\alpha_{e}\left(\frac{\partial^{2}T}{\partial x^{2}}+\frac{\partial^{2}T}{\partial y^{2}}\right),\;$$

where $\left(u,v\right)\;$ denotes the components of velocity along the $\left(x,y\right)\;$ directions and (β, μ, ρ, $\alpha_{e},p,$ D) denotes the Casson parameter, kinematic viscosity, fluid density, thermal diffusivity, pressure, and diffusion coefficient, respectively. $\Lambda =\left(\Lambda_{x},{\;\Lambda }_{y}\right)$ denotes the force index resulting from the magnetic field.

The force index generated in consideration of the Lorentz force and by using the Boussinesq approximation, temperature, and concentration gradients are stated as follows:

(6) $$\Lambda_{x}=\sigma B_{0}^{2}\left(vsin\gamma cos\gamma -usin^{2}\gamma \right)$$

(7) $$\Lambda_{y}=\sigma B_{0}^{2}\left(usin\gamma cos\gamma -{vcos}^{2}\gamma \right)+\rho g\left[\beta_{T}\left(T-T_{c}\right)+\beta_{c}\left(c-c_{c}\right)\right]$$

where, respectively, $\beta_{T}$ and $\beta_{c}$ represent the thermal and solutal expansions.

### 2.3. Boundary Conditions

*n* displays the normal vector on the boundary.

(8) $$u=0,\;\;v=0,\;\;T=T_{h},\;\;c=c_{h}\;\;\left(Hot\;side\right)\;$$

(9) $$u=0,\;\;v=0,\;\;T=T_{c},\;\;c=c_{c}\;\;\left(Cold\;side\right)$$

(10) $$u=0,\;\;v=0,\;\;\frac{\partial T}{\partial n}=\frac{\partial c}{\partial n}=0,\;\;\left(Remaining\;walls\right)$$

The governing, equations are transformed into non-dimensional form using the following parameters.

(11) $$\left(X^{*},Y^{*}\right)=\frac{\left(x,,y\right)}{L},\;\left(U^{*},V^{*}\right)=\frac{\left(u,,v\right)L}{\alpha f},\;P^{*}=\frac{pL^{2}}{\rho \alpha^{2}},\;\;\theta^{*}=\frac{T-T_{c}}{T_{h}-T_{c}},\;\;C^{*}=\frac{c-c_{c}}{c_{h}-c_{c}}$$

(12) $$\alpha_{e}=\frac{k_{e}}{{\left(\rho c_{p}\right)}_{f}},\;Ra=\frac{\rho^{2}\beta_{T}gL^{3}\Delta TPr}{\upsilon^{2}},\;Le=\frac{\alpha_{e}}{D},\;\;Ha=BL\sqrt{\frac{\mu }{\sigma }},\;\;Pr=\frac{\nu }{\alpha }.$$

The governing equations are represented non-dimensionally as follows:

(13) $$\frac{\partial U^{*}}{\partial X^{*}}+\frac{\partial V^{*}}{\partial Y^{*}}=0$$

(14) $$\left(U^{*}\frac{\partial U^{*}}{\partial X^{*}}+V^{*}\frac{\partial U^{*}}{\partial Y^{*}}\right)=-\frac{\partial P^{*}}{\partial X^{*}}+Pr\left(1+\frac{1}{\beta }\right)\left(\frac{\partial U^{*}}{\partial X^{*}}+\frac{\partial U^{*}}{\partial Y^{*}}\right)+\Lambda_{X^{*}}$$

(15) $$\;\;\left(U^{*}\frac{\partial V^{*}}{\partial X^{*}}+V^{*}\frac{\partial V^{*}}{\partial Y^{*}}\right)=-\frac{\partial P^{*}}{\partial Y^{*}}+Pr\left(1+\frac{1}{\beta }\right)\left(\frac{\partial V^{*}}{\partial X^{*}}+\frac{\partial V^{*}}{\partial Y^{*}}\right)+\Lambda_{Y^{*}},$$

(16) $$U^{*}\frac{\partial \theta^{*}}{\partial X^{*}}+V^{*}\frac{\partial \theta^{*}}{\partial Y^{*}}=\frac{\partial^{2}\theta^{*}}{\partial X^{*}{}^{2}}+\frac{\partial^{2}\theta^{*}}{\partial Y^{*}{}^{2}}$$

(17) $$U^{*}\frac{\partial C^{*}}{\partial X^{*}}+V^{*}\frac{\partial C^{*}}{\partial Y^{*}}=\frac{1}{Le}\left(\frac{\partial^{2}C^{*}}{\partial X^{*}{}^{2}}+\frac{\partial^{2}C^{*}}{\partial Y^{*}{}^{2}}\right),$$

where

(18) $$\Lambda_{X^{*}}=PrHa^{2}\left(V^{*}sin\gamma cos\gamma -U^{*}sin^{2}\gamma \right)$$

(19) $${\;\Lambda }_{Y^{*}}=PrHa^{2}\left(U^{*}sin\gamma cos\gamma -V^{*}cos^{2}\gamma \right)+RaPr\left(\theta^{*}+NC^{*}\right)$$

The associated dimensionless boundary conditions are

(20) $$U^{*}=0,\;\;V^{*}=0,\;\;\theta^{*}=1,\;\;C^{*}=1\;\;\left(\mathrm{Hot},\;\mathrm{side}\right)$$

(21) $$U^{*}=0,\;\;V^{*}=0,\;\;\theta^{*}=0,\;\;C^{*}=0\;\;\left(\mathrm{cold},\;\mathrm{side}\right)$$

(22) $$U^{*}=0,\;\;V^{*}=0,\;\;\frac{\partial \theta^{*}}{\partial n}=\frac{\partial C^{*}}{\partial n}=0\;\;\left(\mathrm{Remaining}\;\mathrm{walls}\;\right)$$

The following are definitions of mathematical relations for local and average Nusselt and Sherwood numbers and:

(23) $$Nu_{local}={\left(-\frac{\partial \theta^{*}}{\partial X^{*}}\right)}_{X^{*}=0}$$

(24) $$Sh_{local}={\left(-\frac{\partial C^{*}}{\partial X^{*}}\right)}_{X^{*}=0}$$

(25) $$Nu_{avg}=\frac{1}{S}\int_{S}^{1}Nu_{local}\;dS$$

(26) $$Sh_{avg}=\frac{1}{S}\int_{S}^{1}Sh_{local}\;dS.$$

Additionally, the total kinetic energy is stated as follows:

(27) $$K.E=\frac{1}{2}\int_{\Omega }\|U^{2}\|\;d\Omega$$

where $U=\left(U^{*},\;V^{*}\right)$ is the velocity vector.

## 3. Solution Methodology

**Figure 2:**
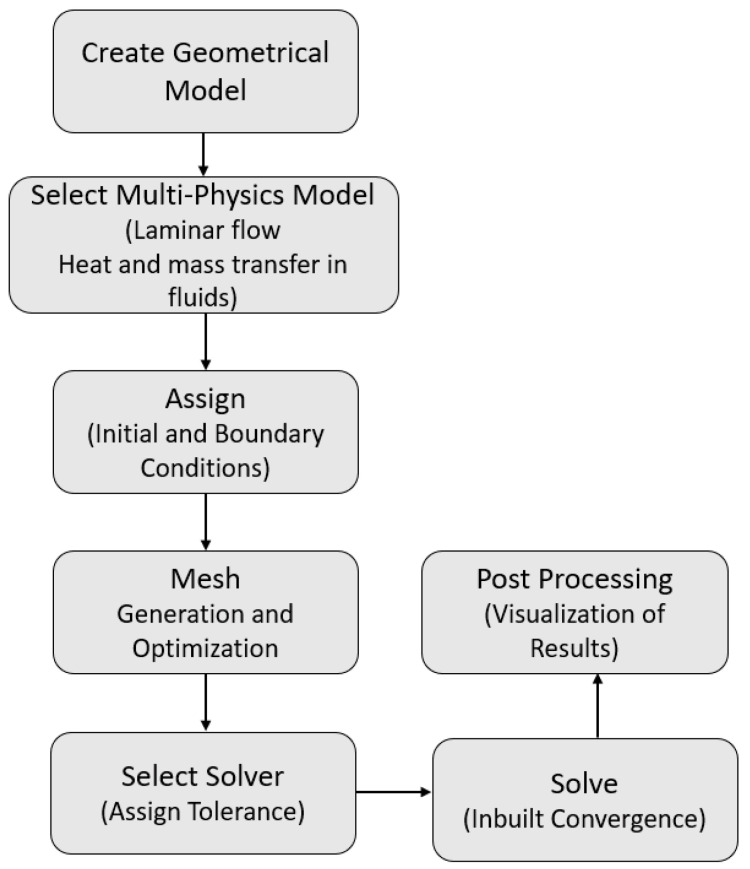
Illustration of the steps involved in COMSOL Multiphysics software.

By using exact approaches, fluid flow behavior in non-confined boundaries can be managed with ease, but using conventional techniques to extract the solution in a closed enclosure with various types of obstacles might be challenging. Therefore, to describe their findings, the majority of researchers use numerical schemes, and the most generous techniques are FDM, FEM, and FVM. Among the aforementioned numerical approaches, the finite element method is one of the most flexible since it can readily simulate complicated and irregular shapes by discretizing the given domain with finite elements. So, in view of complexity of current physical problems, a commercial software known as COMSOL Multiphysics in the latest version 5.6 is used. The main steps involved during the capitalization of the software are represented in Figure 2. 

**Figure 3:**
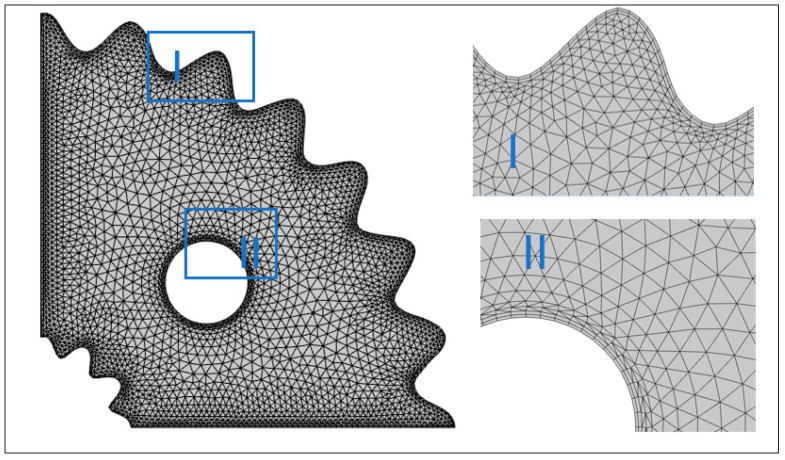
A computational grid at finer-level mesh.

**Figure 4:**
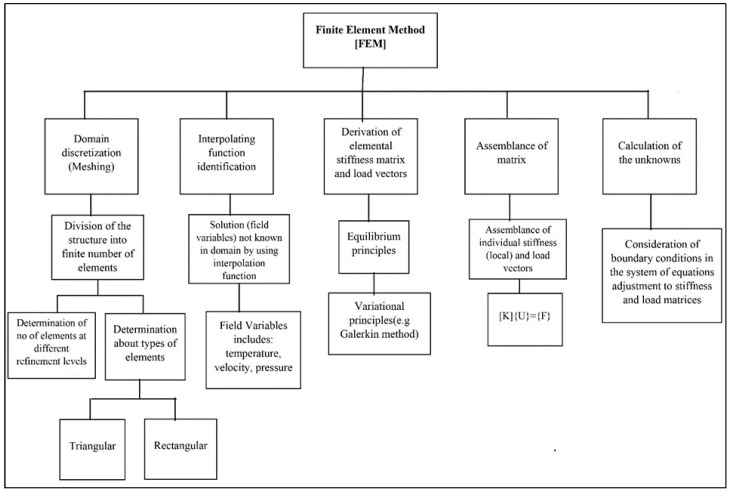
Flowchart for the finite element approach.

As a result, while computing velocity and temperature, we used stable quadratic elements, while approximating pressure using linear elements. In the current pagination, a hybrid finite element mesh composed of rectangular and triangular components is used. The coarse grid-level computational mesh is shown in Figure 3, and the associated degrees of freedom at further levels of refinement are displayed in Table 1. The finite element method’s steps are shown in Figure 4. For the purpose of linearizing non-linearized expressions in FEM, Newton’s technique is utilized, and the resulting linear system of equations is solved using a direct solver that relies on elimination and a unique rearrangement of the unknowns. For the non-linear iterations, the following convergence condition is established:

$$\left|\frac{\chi^{n+1}-\chi^{n}}{\chi^{n+1}}\right|<10^{-6}$$

where the general solution component is represented by the symbol χ.

In the finite element method, the basic step to resolve the problem solution is the conversion of complex domains into small sub-domains called elements. To approximate field variables in these elements, we use different types of interpolating functions. In this work, pressure is approximated by linear function, and other distributions (velocity, temperature, and mass) are approximated by quadratic elements. In addition, hp refinement is obliged for meshing. There are two types of convergences in finite element analysis, which are explained below:

1. The first one is the convergence of solutions, which explains how the algorithm inside the solver may find a problem solution that is accurate and stable enough. In the finite element method, at last, we attain systems of equations which are solved by Paradiso built-in Software in the backup of COMSOL, so a convergent solution is attained when iterations are performed.
2. The second sort of convergence is mesh convergence, which entails systematically reducing the size of elements close to areas of high gradients in order to obtain more precise findings.

In COMSOL, we applied adaptive meshing and quantities of physical interest such as Nusselt and Sherwood numbers, which are computed at different refinement levels. From the table drawn for grid the convergence test (Table 2), which describes variation in heat and mass flux at different refinement levels, no deviation in quantities at level 8 and 9 is shown. The FEA grid convergence is a highly essential test to assure the credibility of work. 

### 3.1. Grids, Convergence 

Grid convergence tests are carried out at different grid levels to demonstrate the validity of capitalized numerical schemes and by fixing $\beta =1,\;Ha=25,\;Le=2.5,\;Pr,=6.8,\;\mathrm{and}\;Ra=10^{5}$, which are revealed in Table 2. Average heat and mass fluxes are computed for this purpose, and an estimate of kinetic energy is also made. It can be shown that the values of the engineering interest quantities described above are consistent at levels 8 and 9.

**Table 2:** Study of grid convergence for average mass and heat fluxes.

Grid	$Nu_{avg}\;$	$Sh_{avg}$	K.E.
1	8.1634	5.6851	192.96
2	8.3042	5.8191	175.23
3	8.7013	5.8818	171.68
4	9.2243	5.9220	166.59
5	9.5047	5.9208	166.28
6	9.7831	5.9269	166.41
7	10.755	5.9313	165.48
8	11.6091	5.9204	164.88
9	11.6095	5.9204	164.85

### 3.2. Validation of Results

By computing the average Nusselt number against the Casson fluid parameter β and fixing $Ha=25,\;Le,\;2.5,\;Pr=6.8,\;\mathrm{and}\;Ra=10^{5}$, as given in Table 3, the results are assured by comparing them with those reported by Hussain et al. [45]. A perfect match is discovered among the outcomes using the obtained data. 

.

## 4. Results and Discussion

This section is provided to understand the results based on stream lines, isotherms, and isoconcentration patterns against different parameters such as $\left(\beta \right)$, $\left(Ra\right)$, $\left(Le\right),$ and $\left(Ha\right)$. In addition, measurements are conducted for kinetic energy as well as mass and heat fluxes. Additionally, some parametric variables, i.e., heat, capacity ($c_{p}=1)$, isotropic thermal conductivity (κ=1), density $\left(\rho =1\right),$ and ratio-specific heat $\left(\gamma =1\right),\;$ are given particular values in order to provide flow distribution variations.

**Figure 5:**
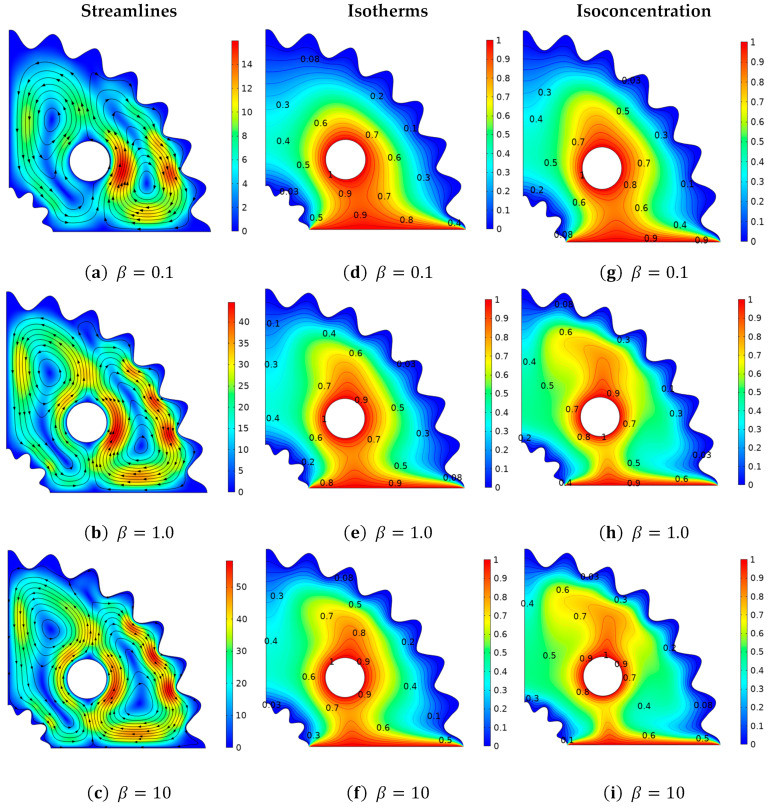
Deviation of (**a**–**c**) stream lines, (**d**–**f**) isotherms, and (**g**–**i**) isoconcentration with respect to the Casson parameter (*β*).

Figure 5 illustrates the influence of the Casson parameter $\left(\beta \right)$ on momentum, temperature, and concentration distribution. The changes in velocity distribution via stream lines against $\left(\beta \right)$ is exclusively discussed in Figure 5a–c. In this instance, it is noted that greater values of the non-Newtonian parameter result in resistance to fluid motion. That is the fact that the velocity profile and the thickness of the boundary layer decrease for greater values of $\left(\beta \right)$. Figure 5d–f reveals the change in the thermal field in relation to the Casson parameter $\left(\beta \right)$. It is revealed that isotherms at $\left(\beta \right)$=10 isotherms magnitude exhibit greater. response because, at higher values of $\left(\beta \right)$ fluid velocities, average kinetic, energy increases and the temperature profile exhibits a positive trend. It is significant to note that the installation of a localized heat source at the bottom wall and the creation of convective thermal potential cause heat to move from the lower portion of the enclosure to the higher portion. The effect of the Casson fluid parameter $\left(\beta \right)$ on the concentration field is revealed in Figure 5d–f. As can be observed, when the $\left(\beta \right)$ factor is less, less fluid particle dispersion is produced, which improves fluid concentration as a result. However, at increasing Casson magnitudes, the fluid exhibits more Newtonian fluid behavior, and its viscosity decays, causing more fluid disturbance and a decrease in the concentration field, since the base wall and circular cylinder in the current work are uniformly concentrated to produce buoyancy forces. According to the illustration, viscosity is maximum at lower magnitudes of $\left(\beta \right)$ and fluid molecules gather close to the base wall. In addition, the squeezing of the regions at higher magnitudes of $\left(\beta \right)$ is observed.

**Figure 6:**
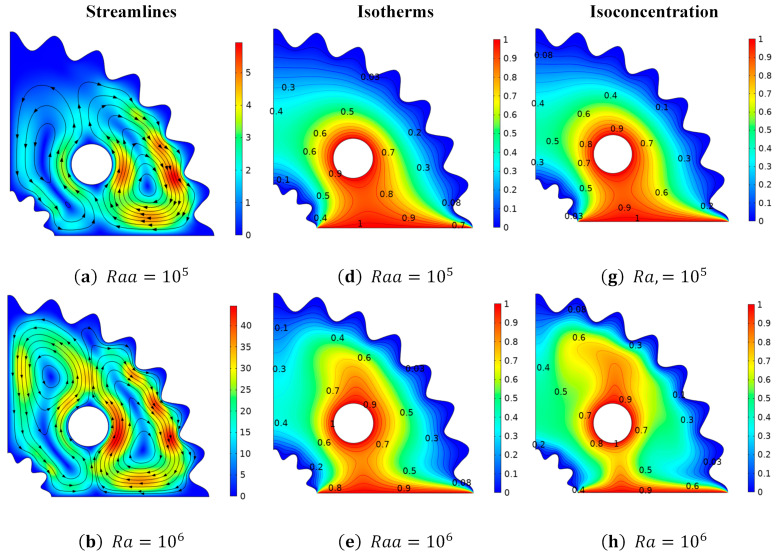

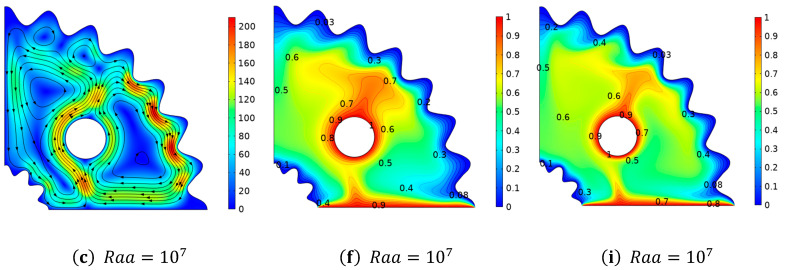
Deviation of (**a**–**c**) stream lines, (**d**–**f**) isotherms, and (**g**–**i**) isoconcentration with respect to Rayleigh number (*Ra*).

Changes in stream lines, isotherms, and isoconcentrations relative to (Ra) varying from $Ra=10^{5}-10^{7}$ are shown in Figure 6. In Figure 6a–c, it is evident that the amplitude of the stream function, as interpreted by the stream lines, mounts. against the growing magnitude of the Rayleigh number (Ra). It is supported by the mathematical relationship that exists for the dimensionless Rayleigh number coefficient, i.e., $Ra=\frac{\rho^{2}\beta_{T}gL^{3}\Delta TPr}{\upsilon^{2}}$. This expression shows that fluid viscosity reduces with an increase in (Ra), since (Ra) has an inverse relationship with, viscous forces. It is important to note that four vortices arise and that stream line deviations appear at larger magnitudes of (Ra). Additionally, it appears that the fluid is moving in the upper and lower halves in the opposing directions. Figure 6d–f uses isotherm patterns to express the change in temperature distribution relative to (Ra). It has been noted that when the magnitude of (Ra) increases, fluid temperature rises, which is supported by isothermals contours patterns. This fact is supported by the relationship that shows that when (Ra) increases, the temperature differential between, hot and cold regions widens and thermal buoyancy forces are generated. Therefore, the region above the cylinder widens and the maximum diffusion of the temperature distribution is seen at Ra = $10^{7}$. Figure 6 reveals the variation in the concentration profile against Rayleigh number (Ra). It is adhered that fluid concentration is higher at lower magnitudes of (Ra) than at higher magnitudes of (Ra).

The cause of this behavior is that fluid accumulates, and the concentration field rises for lower values of (Ra), i.e., Ra = $10^{7}$, where a low solutal convective potential is generated by a smaller concentration difference.

**Figure 7:**
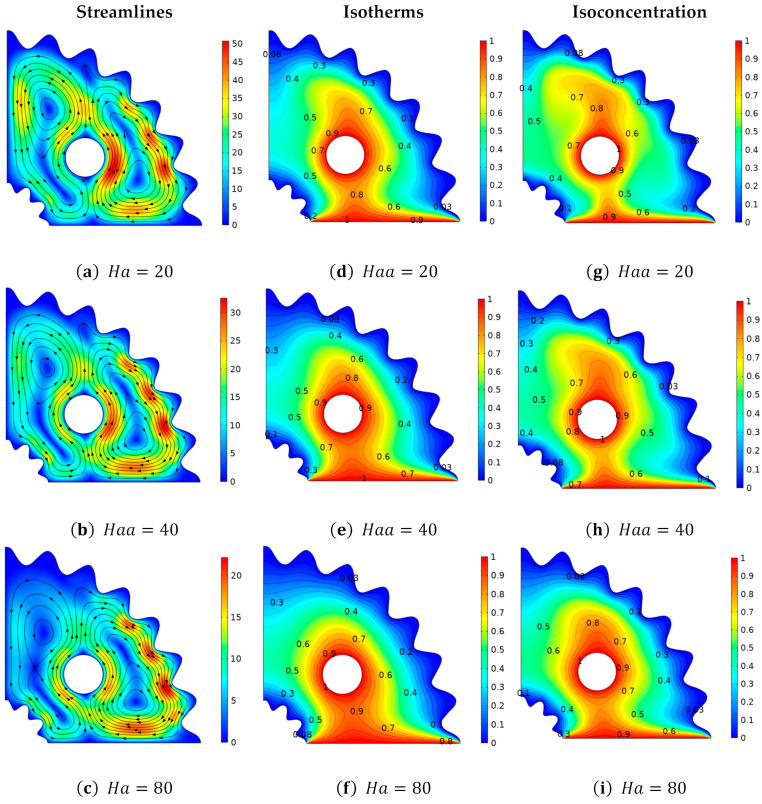
Deviation of (**a**–**c**) stream lines, (**d**–**f**) isotherms, and (**g**–**i**) isoconcentration with respect to the Hartmann number (*Ha*).

**Figure 8:**
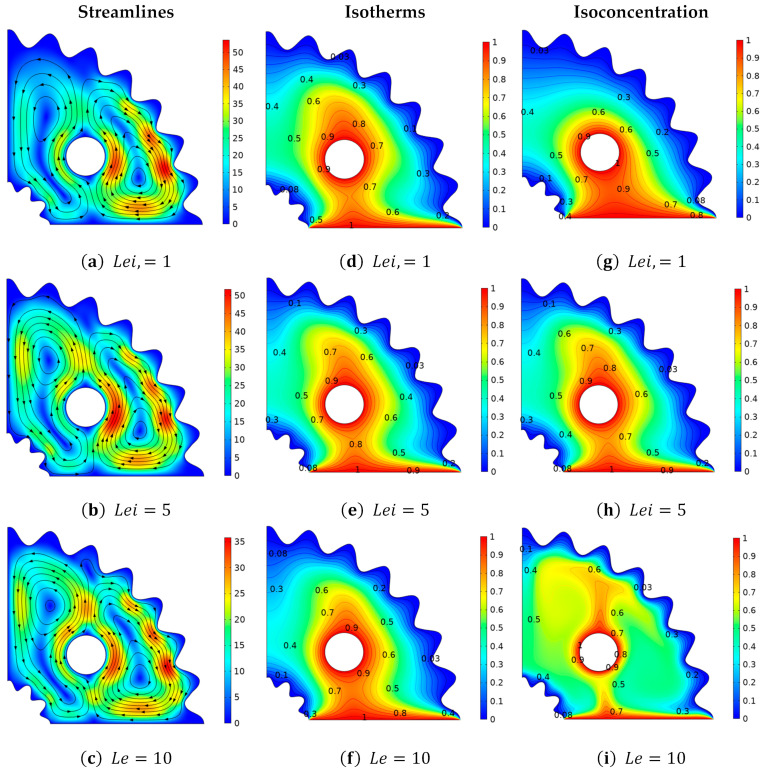
Deviation of (**a**–**c**) stream lines, (**d**–**f**) isotherms, and (**g**–**i**) isoconcentration with respect to the Lewis number (*Le*).

Figure 7 depicts the change in velocity, temperature, and concentration distributions when the magnitude of the magnetic field parameter (Ha) is increased. Since, the Hartmann number (Ha) is implicated in the current investigation due to the inclusion of a magnetic field, which plays a role, in reducing the velocity profile and making the flow regime, laminar. The flow behavior of Casson fluid in curved corrugated structures against the deployment of the Hartmann number (Ha) is explained in Figure 7a–c. At Ha = 80, the velocity is at its lowest magnitude, while at Ha = 20, the velocity distribution is at its highest value. It is because the opposing Lorentz force produced by the high Hartmann number (Ha) causes resistance, to the fluid and stops the motion. Figure 7d–f depicts a variation in thermal distribution based on isothermal contours vs. Hartmann number. The Hartmann number (Ha) indicates the ratio of $Ha=BL\sqrt{\frac{\mu }{\sigma }}$, in which it can be shown that as the viscosity of a fluid increases against (Ha) due to which the fluid’s average kinetic energy decreases, which has the effect of causing a decay in the temperature distribution. Figure 7g–i shows that the concentration distribution improves as the Hartmann number (Ha) increases. It is so because as (Ha) increases, viscosity improves, due to which fluid concentration rises as a result. Additionally, with Ha = 80, a larger isoconcentration region is generated. In Figure 8, we looked at the distributions of momentum, temperature, and concentration as a function of Lewis number (Le), which varies from “01 to” 10. Figure 8a–c analyzes velocity behavior change in relation to Lewis number (Le). Since (Le) has no direct relationship to momentum diffusivity, no significant, changes in momentum distribution are seen against it. The impact of the Lewis numbers (Le) on isothermal contours are shown in Figure 8d–f by fixing, *Pr* = 6.8, *Ha* = 25, β=1, $Ra=10^{5}$, *N* = 1, and γ = 1. Similar to the velocity profile, no discernible change in the temperature distribution is seen against Lewis number (Le). Figure 8g–i examines the positive trend in the amplitude of the mass flux versus (Le). Given that the Lewis number is the ratio of the thermal-to-mass diffusivities., increasing (Le) causes the thermal diffusion to rise, while causing the mass diffusivity to fall. As a result, the optimized region is obtained at Le = 1, where there is less mass dispersion, while the isoconcentration region is thinner at Le = 10. Near the hot surface, a large concentration of fluid is present.

Table 4 shows numerical information about changes in the average Nusselt number $\left(Nu_{avg}\right)$ and average Sherwood number ($Sh_{avg}$) in relation to the Hartmann number, and Casson fluid, parameter (β) by fixation, *Pr* = 6.8, *Ra* = 10^5^, *Le* = 2.5, *N* = 1, and γ = 30°. As can be shown, the highest mean Nusselt number and Sherwood number, with magnitudes of 13.813 and 11.395, respectively, were found at *β* = 10 and Ha = 0. Because there is no magnetic field at Ha = 0, there are no resistive forces, as a result, velocity and kinetic energy are both very high, and temperature flux is enhanced. Average heat and mass fluxes are growing because the fluid is approaching Newtonian behavior when the Casson parameter (*β*) is increased. As a result, the fluid’s viscosity is decreasing, causing kinetic energy to surpass and the two flux rates listed above to increase. However, average heat and mass fluxes show a different pattern when measured against the Hartmann number. This fact is demonstrated by the generation of resistive forces in the flow domain as a result of an increase in (Ha) and a decrease in kinetic energy.

The averages kinetic energy varies for several Hartmann numbers, and Casson parameter (*β*) values, as seen in Table 5. The findings showed that the kinetic energy was enhanced 26.5 times at *β* = 10 and Ha = 0 compared with *β* = 1.0 and Ha = 0. It can be observed that as the Casson parameter (*β*) is increased, Casson fluid begins to behave like Newtonian fluid, which results in a decrease in fluid viscosity. This reduction in viscosity increases fluid velocity while also increasing kinetic energy. In contrast, decreasing aptitude in kinetic energy is observed versus (Ha) because of the creation of Lorentz forces, which provides resistance to fluid particle motion and restricts increases in kinetic energy.

## 5. Concluding Remarks

The current communication examines the Double-Diffusive Natural, Convection (DDNC) regime in Casson fluid flow in a curved corrugated enclosure, for uniform temperature and concentration distributions and by putting a heated and concentrated cylinder. The mathematical representation of the problem is carried out in the form of a dimensionless partial differential system by capitalizing the governing law. The finite element approach is used to perform numerical simulations. The accompanying momentum, temperature, and concentration distributions vary in response to steam lines, isothermal, and isoconcentration patterns. Engineering, quantities such as kinetics energy, local Nusselt, and Sherwood number are also assessed against dimensionless involved physical parameters. The following are the key findings of the current study:

- By, increasing the Lewis number, the mass distribution decreases, as justified by the isoconcentration pattern.
- The temperature distribution improves while the concentration profile deteriorates as the Rayleigh number increases.
- Intensification of kinetic energy is noticed against the Hartmann number, while the opposite feature is exhibited against $\left(\beta \right)$.
- Heat and mass flux coefficients decrease in relation to the Hartmann number (Ha).
- Heat and mass flux distributions exhibit an upsurging tendency when measured against the Casson parameter $\left(\beta \right)$.
- Because of the heat and concentrated circular obstacle, considerable heat and mass, diffusions are detected in its surroundings.
- A significant factor in convective heat and mass transmission is the Rayleigh number (Ra).

## Figures and Tables

**Table 1:** Mesh statistics for different levels of refinement.

Grid	No. Elements	Degree of Freedoms
1	390	2607
2	552	3630
3	878	5643
4	1476	9284
5	2130	13,189
6	3388	20,504
7	8092	48,224
8	20,002	117,161
9	27,678	159,379

**Table 3:** Comparison of results with the published work of Hussain et al. [45] for various values of β.

β	Hussain et al. [45]	Present
0.1	2.389863	2.390235
1.0	3.811945	3.811682
5.0	4.275872	4.275113
10	4.355561	4.354701

**Table 4:** Changes in the average Nusselt and Sherwood numbers in relation to the Casson parameter (β) and the Hartmann number (Ha).

$Nu_{avg}$				$Sh_{avg}$		
Ha	β=0.1	β=1	β=10	β=0.1	β=1	β=10
0	10.208	12.511	13.813	7.2124	10.467	11.395
25	10.133	11.609	12.312	7.6856	9.9592	10.721
50	10.021	10.565	10.802	7.6880	9.0789	9.5956
75	9.9600	10.134	10.207	7.6435	8.5659	8.8763
100	9.9301	9.9959	10.020	7.5533	8.1425	8.3254

**Table 5:** Average kinetic variation for different Hartmann numbers (Ha) and Casson parameters (*β*).

Kinetic Energy			
Ha	β=1	β=5	β=10
0	25.956	327.11	661.12
25	19.325	164.88	268.93
50	10.831	57.068	80.792
75	6.0996	23.557	31.014
100	3.4479	10.630	13.474

## Data Availability

All data is available in the manuscript.

## Nomenclature

𝑔	Gravitational acceleration	$B_{0}$	Magnetic field strength
𝑦	Vertical coordinate	μ	Dynamic viscosity
𝑆ℎ	Sherwood number	𝑇	Temperature
𝑣	y-coordinate velocity	𝑃	Fluid pressure
𝐿𝑒	Lewis number	$k_{e}$	Thermal conductivity
𝑃𝑟	Prandtl. number	$\alpha_{e}$	Thermal diffusivity
𝑥	Horizontal. coordinate	𝑁𝑢	Nusselt number
𝑅𝑎	Rayleigh, number	$C_{p}$	Specific heat
*Ha*	Hartmann number	𝑢	x-coordinate velocity
𝑐	Concentration		
Greeks Symbols			
ρ	Fluid density	θ	Temperature
𝜷	Casson fluid parameter		
